# Twenty-Year Mortality Trends in Patients with Kidney Disease in Poland with the Use of the Years of Life Lost Measure, 2000–2019

**DOI:** 10.3390/ijerph19052649

**Published:** 2022-02-24

**Authors:** Paulina Paciej-Gołębiowska, Małgorzata Pikala

**Affiliations:** Department of Epidemiology and Biostatistics, Medical University of Lodz, Żeligowskiego 7/9, 90-742 Lodz, Poland; paulina.paciej-golebiowska@umed.lodz.pl

**Keywords:** kidney diseases, years of life lost, mortality, epidemiology, trends, Poland

## Abstract

Due to the significant socioeconomic burden of kidney diseases, we decided to analyse years of life lost (YLLs) from this cause in Poland between the years 2000 and 2019. The standard expected years of life lost (SEYLL) measure was used to calculate the number of YLLs, the value of which was related to the size of the study population and calculated per 100,000 persons (SEYLLp). A time trend analysis was performed using the Joinpoint regression method. In 2000, the number of YLLs for the entire population was 72,795 (SEYLLp = 190.3 years). After a period of minor changes between 2000 and 2011 (increasing at 0.9% per year), the YLL index rapidly declined between 2011 and 2015 (at −15.4% yearly) and then increased in the last years of the study period (at 12.5% yearly). These changes resulted in a decrease in the number of YLLs to 57,278 in 2019 (SEYLLp = 149.2). The deteriorating health status of Poles after 2015 most likely was caused by the aging of the population, as well as the increasing incidence of risk factors, in particular diabetes and arterial hypertension.

## 1. Introduction

Kidney diseases, especially chronic kidney disease (CKD), pose a serious global health problem. It is estimated that approximately 10–11% of the world population have developed one stage of CKD, which corresponds to 500–600 million patients [1]. According to the Global Burden of Disease (GBD) report, in 2017, CKD contributed to 1.2 million deaths and 35.8 million disability-adjusted life years (DALYs) worldwide. Because kidney disease increases cardiovascular risk, it was estimated that another 1.4 million cardiovascular deaths and 25.3 million DALYs indirectly resulted from CKD-related morbidity [2,3]. Furthermore, the 2019 GBD report indicated that CKD was the eighteenth most important cause of DALYs among 369 analysed diseases, whereas, in 1990, it was the twenty-ninth most important cause. Over the period 1990–2019, the percentage of DALYs due to this cause rose by 93.2% (by 6.3% of age-standardised DALYs) [4]. Additionally, an increase of mortality rates was noted—crude death rate (CDR) rose from 11.2 per 100,000 in 1990 to 18.5 per 100,000 in 2019, and standardized death rate (SDR) rose from 16.1 per 100,000 to 18.3 per 100,000, respectively [5].

The DALY measure consists of the number of years of life lost (YLLs) due to premature deaths and the number of years of life with disability (YLDs) related to reduced quality of life due to a disease. This indicator thus attempts to synthesise the effects of morbidity and mortality, facilitating an evaluation of socioeconomic aspects of the analysed diseases [6]. However, GBD revealed that for CKD only approximately 20% of DALYs are the share of YLDs and the vast majority are YLLs. Globally in 1990, 72.5 YLDs per 100,000 and 329.5 YLLs per 100,000 were caused by CKD, whereas in 2019 it was 113.0 and 423.8, respectively [2,5]. Because YLLs are calculated on the basis of data obtained from death certificates, and the number of YLDs is estimated mainly based on a patient’s self-rated health status, YLLs calculations seem more accurate [6,7,8,9].

Bearing in mind the significant socioeconomic burden of kidney diseases, we tried to assess changes in mortality (using CDR and SDR) and the number of YLLs (using SEYLL) from this cause in Poland, between the years 2000 and 2019.

## 2. Materials and Methods

### 2.1. Research Material

The research material was a dataset based on 7,610,524 death certificates of all the inhabitants of Poland who died in the years 2000–2019, provided by the Department of Information of the Central Statistical Office in Poland. In the study, we used information on 85,224 deaths caused by kidney diseases. These were encoded as N00–N29, N39, and Q61, according to the International Statistical Classification of Diseases and Related Health Problems, 10th Revision (ICD-10). Data on deaths due to particular causes is available as Supplementary Materials.

### 2.2. Statistical Analysis

CDR by sex and age were calculated and then standardized with the use of direct method, adopting the structure of standard European population (updated in 2013) as a reference [10]. Data on the size of a population in particular age groups was obtained from the Local Data Bank of the Central Statistical Office in Poland [11]. The CDR and SDR values were calculated per 100,000 population:

(1) $$CDR=\frac{k}{p}\times 100,000$$

where: *k*—number of deaths, *p*—number of inhabitants;

(2) SDR=∑x  m AxS Px∑x  P Sx

where: *m_x_*—age specific death rate at age *x* last birthday in population, *P_x_*—population exposed to the risk of death at age *x* last birthday in the standard population. YLLs were calculated using the Standard Expected Years of Life Lost (SEYLL) measure, described by Christopher Murray and Alan Lopez [7]:

(3) $$SEYLL=\overset{l}{\underset{x=0}{\sum }}d_{x}e_{x}^{*}$$

where: $e_{x}^{*}$—life expectancy, *d_x_*—number of deaths at age *x*, *x*—age at which the person died, l —oldest age in particular population.

While using the SEYLL index, the number of YLLs in the studied population is compared to years lost by the “standard” population. In our study, we implemented a lifetable recommended by the World Health Organization (WHO) as a reference [12].

We also applied the SEYLL per person index (SEYLLp), which is a ratio of SEYLL and the size of the population, calculated per 100,000 inhabitants, and the SEYLL per death index (SEYLLd), being a ratio of SEYLL and the number of deaths due to a particular cause.

Time trend analysis was carried out with the use of the Joinpoint Regression Program, developed by the U.S. National Cancer Institute [13]. Joinpoint regression is a version of linear regression:

y=bx+a

where: *b*—slope coefficient, *a*—y-intercept. *Y* = ln(*z*), *z*—measure evaluated in the study (SDR, SEYLL) and *x*—calendar year.

Time trend is expressed with a broken line, being a sequence of segments joined in points, where the change of the value is statistically significant (*p* < 0.05). To confirm statistical significance, the Monte Carlo Permutation method was applied. For each segment of straight lines, the annual percent change (APC) was estimated, while for the whole study period, the values of the average annual percent change (AAPC) were given, together with the corresponding 95% confidence interval (CI) [14].

### 2.3. Ethics Approval

The Bioethics Committee of the Medical University of Lodz gave consent for the research to be conducted (No. RNN/183/17KE of 13 June 2017).

## 3. Results

**Figure 1:**
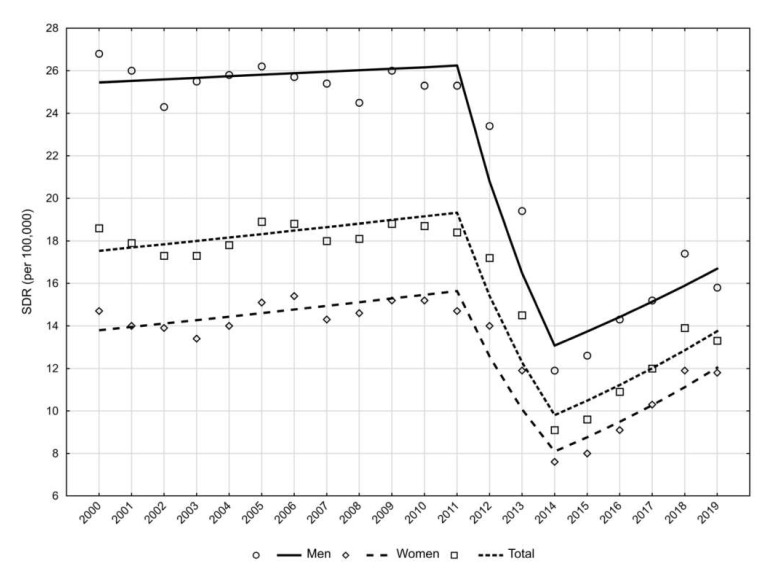
Trends in the standardised death rate (SDR) in patients with kidney disease in Poland from 2000 to 2019 by gender (per 100,000).

Between 2000 and 2019, 85,224 deaths of patients with kidney diseases were recorded in Poland. In 2000, the SDR value for this cause was 18.6 per 100,000 population and remained relatively constant until the year 2011 (APC = 0.9%, *p* < 0.05). Then, in the years 2011–2014, it decreased by 20.2% yearly (*p* < 0.05), but in each subsequent year of the study its value increased at a rate of 7.0% (*p* < 0.05), reaching a value of 13.3 in 2019. Over the analysis period, SDR values were higher in the male group than in the female group (Table 1) (Figure 1).

**Figure 2:**
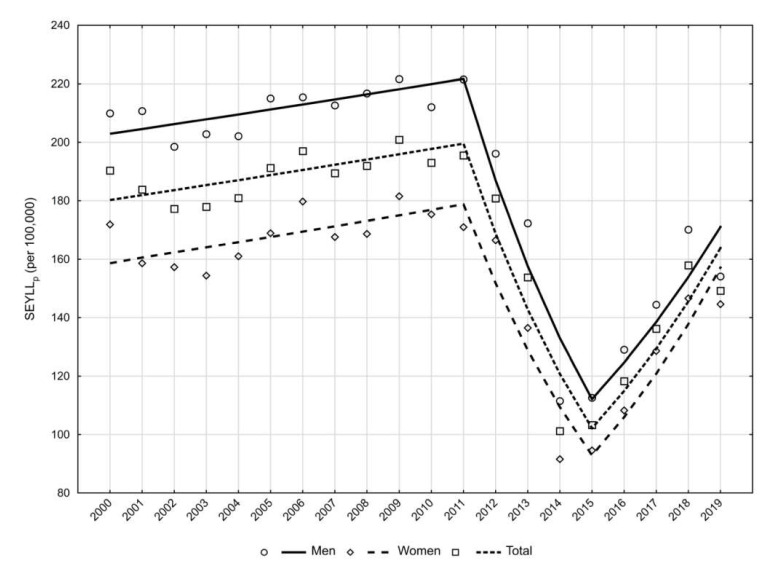
Trends in years of life lost per person (SEYLLp) in patients with kidney disease in Poland between 2000 and 2019 by gender (per 100,000).

The number of YLLs in patients with kidney diseases was 72,795 (SEYLLp = 190.3) in the year 2000, including 38,911 YLLs for men (SEYLLp = 209.9) and 33,884 YLLs for women (SEYLLp = 171.9). The pattern of changes in the SEYLLp coefficients was similar to the SDR trends—a period of minor changes between 2000 and 2011 (APC = 0.9%, *p* > 0.05) was followed by a rapid decline between 2011 and 2015 (APC = −15.4%, *p* < 0.05), and then the value increased again in the last years of the study period (at 12.5% yearly, *p* < 0.05). (Figure 2). AAPC was −0.5% in the whole analysed period (−0.9%, *p* > 0.05 in the male group and −0.1%, *p* > 0.05 in the female group). These changes resulted in a decrease in the SEYLL index to 57,278 in 2019 (SEYLLp = 149.2), including 28,619 years in the male group (SEYLLp = 154.1) and 28,659 years in the female group (SEYLLp = 144.6) (Figure 2).

**Figure 3:**
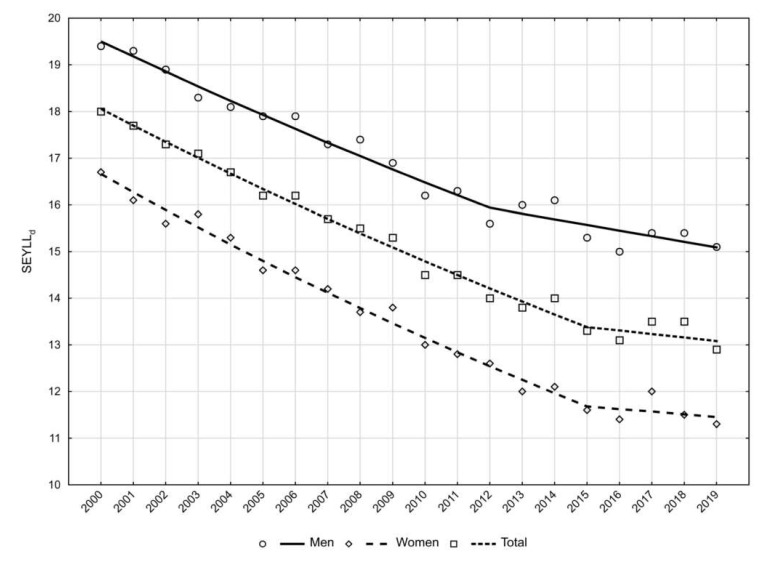
Trends in years of life lost per death (SEYLLd) in patients with kidney disease in Poland from 2000 to 2019.

The inclusion of the SEYLLd index in the study enabled the assessment of how many years of life were lost on average for each person who died with kidney disease. In 2000, SEYLLd was 18.0 years. Throughout the analysed period, its value steadily decreased at an average annual rate of −1.7% (APC for 2000–2015 = −2.0, *p* < 0.05, APC for 2015–2019 = −0.6, *p* > 0.05), and in 2019 it was 12.9 years (Figure 3). In the male group, SEYLLd was 19.4 years in 2000 and 15.1 years in 2019 (AAPC = −1.3%, *p* < 0.05). In the female group, SEYLLd values were lower than in the male group, being 16.7 years in 2000 and 11.3 years in 2019 (AAPC = −2.0%, *p* < 0.05) (Table 2) (Figure 3).

## 4. Discussion

The results of the study showed that the rates of death and indices of years of life lost in patients with kidney disease in Poland significantly decreased over the period 2011–2015; however, in the following years of the study they started to rise again (Figure 1 and Figure 2). The increase of SEYLLp was faster than the increase of SDR (APC = 12.5% vs. 7.0%). Thus, the epidemiological situation worsened, not only due to the rising number of deaths, but also to a slight shift of these deaths to younger age groups. This was also confirmed by the lower rate of decline of SEYLLd in the last years of the study.

For individual patients, the sustained downward trend of SEYLLd is particularly favourable information (Figure 3). An upward trend of SEYLLp with a simultaneous downward trend of SEYLLd is also observed in Poland for some other diseases, e.g., breast cancer or prostate cancer. For these diseases, improving survival rates are the cause [15,16].

In our study, the values of the analysed indices were higher in men than in women, but for both sexes they had a similar pattern of change over time (Table 1 and Table 2). Most studies confirm higher values of mortality rates and YLLs due to kidney diseases in men then in women. The unhealthier lifestyle of men and the damaging effect of testosterone are among causative factors; however, the etiology is complex [17,18,19]. There are also reports on greater all-cause mortality and YLLs in women with kidney failure compared to men [20]. There is still a need to deepen the analysis of health inequalities between the sexes in this regard.

GBD data from 2016 show that, of the European countries, the highest number of YLLs (calculated per 100,000 population) due to chronic kidney disease are recorded in Greece—538, Germany—467 and Austria—429, whereas the average values for the European Union and Poland are 284 and 185, respectively [5]. It should be noted that the GBD report used different ICD-10 codes for the above-mentioned statistics than we did in our study, and they also included four-character codes not available in our database (D63.1, E10.2, E11.2, E12.2, E13.2, E14.2, I12–13.9, N02–N08.8, N15.0, N18–N18.9, Q61–Q62.8) [2].

The factors contributing to the increased mortality from CKD include: (1) population growth and aging of the population, (2) increasing incidence of diabetes and hypertension, which are the most important risk factors for CKD, and (3) limited availability of renal replacement therapy [21,22].

The percentage of elderly people is smaller in Poland than in EU countries, but the dynamics of change in this aspect is noteworthy. According to Eurostat, in 2019, 17.7% of Poles belonged to the group of people aged 65 years or above, while in 2009, the number was 13.5%. In contrast, these percentages were 21.5% and 20.4% for Germany, 18.8% and 17.4% for Austria and 18.5% and 16.6% for Switzerland, respectively, while the average value for the European Union countries (EU-27) was 20.3% in 2019 and 17.4% in 2009 [23].

GBD indicates that among risk factors for CKD are impaired fasting plasma glucose, high blood pressure, high body-mass index, a diet high in sodium, and lead. Diabetes is estimated to be responsible for more than 30% of CKD cases [2], which was also proven by the Polish Nephrology Registry [24]. It is assessed that approximately 7% of Poles, particularly the oldest members of the population, have diabetes [25]. Data from the POLSENIOR epidemiological study conducted in 2011–2013, among people aged 65 years and older, indicate that 23.1% of the subjects are affected by diabetes [26]. However, the prevalence of hypertension among Poles is even higher. The NATPOL 2011 study confirmed its incidence in 32.5% of people aged 18–79 years, but the highest prevalence was observed in respondents aged 60–79 years, i.e., in 67.8% [27]. The POLSENIOR study showed that hypertension was present in 78.2% of women and 70.1% of men aged 65 years or older [28]. Globally, approximately 462 million people suffer from diabetes, which accounts for 6.28% of the total population. With regards to people aged 70 years or above, as many as 22% are affected by this disease [29]. Hypertension already affects more than 30% of people worldwide [30]. Activities aimed at the early detection of these diseases and prevention of organ complications need to be intensified at the level of primary care.

Limited access to renal replacement therapy may be another cause of worsening mortality rates due to kidney diseases. However, this does not seem to be the case in Poland. The availability of renal replacement therapy in Poland is similar to that in other EU countries. The total number of people treated in 2019 with haemodialysis, peritoneal dialysis or kidney transplantation was 888 per 1,000,000 Polish population (to compare, it was 370 per 1,000,000 in 2002 and 694 per 1,000,000 in 2010). However, the number of patients waiting for kidney transplantation has recently decreased (in 2019, 5.4% of dialysis patients were on waiting lists, 6.0% in 2018, and 13% in 2017). According to the Polish Nephrology Registry, advanced age of the patients and accompanying multimorbidity disqualify them from the procedure [24].

A survey on the opinions of patients treated with renal replacement therapy, conducted by the European Kidney Patient’s Federation in 2010–2011, confirmed that Polish patients were satisfied with the information they received regarding transplantation and hospital haemodialysis. Furthermore, almost 75% of patients claimed that they knew where to report their dissatisfaction with treatment, and this was one of the best results observed in Europe. Additionally, more Polish patients were reposted to receive adequate counselling or rehabilitation than an average patient in Europe [31].

The satisfactory epidemiological situation observed in Poland before 2015 could also have been contributed to by the Early Detection of Kidney Diseases Programme implemented between 2003 and 2011. Within this campaign, more than 100 meetings in a form of workshops and conferences were held and they were attended by more than 5000 physicians of various specialties. Furthermore, a lot of prophylactic and educational programmes for patients were carried out in the largest Polish cities [1]. Activities promoting knowledge of kidney diseases, both among physicians and the general public, supported by early diagnostic programmes are recognized factors that reduce the burden of CKD [2,32].

### Limitations of the Study

The reporting of deaths in Poland is 100% and data on deaths are considered the most reliable source of information on the health status of the population However, the use of death certificates as research material is a certain limitation of the study. Integrity is maintained on condition that the underlying cause of death is properly identified by physicians, and then properly assigned to the ICD-10 code. Data from 2012 indicate that in Poland almost 30% of death certificates are assigned the wrong ICD-10 code, but in more than 70% this concerns the determination of cardiological causes of death [33,34].

Another limitation of the study is the difficulty in comparing the obtained results with other works due to: (1) inclusion of various ICD-10 codes or (2) different methodologies applied for estimating the number of YLLs.

In our work, we used the SEYLL index for which the number of YLLs in the studied population is compared to the years lost by the “standard” population. The WHO recommends as standard the lifetables, which are compiled on the basis of the lowest mortality rates for each age group in countries with a population over 5 million [12]. However, it is also possible to use other reference values (e.g., Coale–Demeny model life tables) or even other indices. The simplest is the Potential Years of Life Lost (PYLL) measure, computed as a difference between an arbitrary limit of life expectancy (usually between 60 and 85 years) and the age of people at the time of death. Its most important negative implication is that it ignores the benefits of activities aimed at the oldest members of the population. Another measure is Period Expected Years of Life Lost (PEYLL), for which local life tables are used for calculation. Due to differences in local life expectancy, its applicability to comparisons between populations and even of the same population over time, is limited.

## 5. Conclusions

After favourable downward trends of the rates of death and indices of years of life lost in patients with kidney diseases, which were observed in Poland in the period 2011–2015, disturbing upward trends of the above-mentioned measures were observed in the last years of research. Most likely, this is caused by the aging of the population, as well as the increasing incidence of risk factors, in particular diabetes and arterial hypertension.

The study indicated that the problem of premature mortality due to kidney diseases affects men to a greater extent than women, but the data from the literature are not conclusive. Further research should be focused on the search for a particularly vulnerable socioeconomic group, to which it will be possible to prioritize activities aimed at reversing the current unfavourable trend.

## Figures and Tables

**Table 1:** Death rates in patients with kidney diseases in Poland between 2000 and 2019 by gender.

Year	CDR			SDR		
	General	Men	Women	General	Men	Women
2000	10.5	10.8	10.3	18.6	26.8	14.7
2001	10.4	10.9	9.9	17.9	26.0	14.0
2002	10.3	10.5	10.1	17.3	24.3	13.9
2003	10.4	11.1	9.8	17.3	25.5	13.4
2004	10.8	11.1	10.5	17.8	25.8	14.0
2005	11.8	12.0	11.6	18.9	26.2	15.1
2006	12.2	12.0	12.3	18.8	25.7	15.4
2007	12.0	12.3	11.8	18.0	25.4	14.3
2008	12.4	12.4	12.3	18.1	24.5	14.6
2009	13.2	13.1	13.2	18.8	26.0	15.2
2010	13.3	13.1	13.5	18.7	25.3	15.2
2011	13.5	13.6	13.4	18.4	25.3	14.7
2012	12.9	12.5	13.2	17.2	23.4	14.0
2013	11.1	10.8	11.4	14.5	19.4	11.9
2014	7.2	6.9	7.5	9.1	11.9	7.6
2015	7.8	7.4	8.2	9.6	12.6	8.0
2016	9.1	8.6	9.5	10.9	14.3	9.1
2017	10.1	9.4	10.7	12.0	15.2	10.3
2018	11.9	11.0	12.7	13.9	17.4	11.9
2019	11.5	10.2	12.8	13.3	15.8	11.8

CDR—Crude Death Rate (per 100,000); SDR—Standardised Death Rate (per 100,000).

**Table 2:** Years of life lost in patients with kidney disease in Poland from 2000 to 2019 by gender.

Year	SEYLLp			SEYLLd		
	General	Men	Women	General	Men	Women
2000	190.3	209.9	171.9	18.0	19.4	16.7
2001	183.8	210.7	158.6	17.7	19.3	16.1
2002	177.2	198.5	157.3	17.3	18.9	15.6
2003	177.9	202.8	154.4	17.1	18.3	15.8
2004	180.9	202.1	161.0	16.7	18.1	15.3
2005	191.2	215.0	168.9	16.2	17.9	14.6
2006	197.0	215.4	179.7	16.2	17.9	14.6
2007	189.4	212.6	167.6	15.7	17.3	14.2
2008	191.9	216.7	168.7	15.5	17.4	13.7
2009	200.9	221.6	181.5	15.3	16.9	13.8
2010	193.0	212.0	175.3	14.5	16.2	13.0
2011	195.5	221.5	171.0	14.5	16.3	12.8
2012	180.8	196.1	166.5	14.0	15.6	12.6
2013	153.8	172.3	136.5	13.8	16.0	12.0
2014	101.2	111.5	91.6	14.0	16.1	12.1
2015	103.3	112.6	94.6	13.3	15.3	11.6
2016	118.3	129.0	108.3	13.1	15.0	11.4
2017	136.2	144.4	128.6	13.5	15.4	12.0
2018	157.9	170.1	146.6	13.3	15.4	11.5
2019	149.2	154.1	144.6	12.9	15.1	11.3

SEYLLp—Standard Expected Years of Life Lost per person (per 100,000); SEYLLd—Standard Expected Years of Life Lost per death.

## Data Availability

The data presented in this study are available on request from the corresponding author.

## Supplementary Materials

The following are available online at https://www.mdpi.com/article/10.3390/ijerph19052649/s1, Figure S1: Number of deaths in patients with kidney disease in Poland in 2000-2019 according to cause (ICD-10 codes).
