# Correction: Paciej-Gołębiowska et al. Twenty-Year Mortality Trends in Patients with Kidney Disease in Poland with the Use of the Years of Life Lost Measure, 2000–2019. *Int. J. Environ. Res. Public Health* 2022, *19*, 2649

**DOI:** 10.3390/ijerph20075355

**Published:** 2023-03-31

**Authors:** Paulina Paciej-Gołębiowska, Ilona Kurnatowska, Irena Maniecka-Bryła, Małgorzata Pikala

**Affiliations:** 1 Department of Epidemiology and Biostatistics, Medical University of Lodz, Żeligowskiego 7/9, 90-742 Lodz, Poland; 2 Department of Internal Medicine and Transplant Nephrology, Medical University of Lodz, Kopcinskiego 22, 90-549 Lodz, Poland

## Addition of Authors

Ilona Kurnatowska and Irena Maniecka-Bryła were not included as authors in the original publication [1]. The corrected Author Contributions statement appears here. The authors state that the scientific conclusions are unaffected. This correction was approved by the Academic Editor. The original publication has also been updated.

## Addition of an Affiliation

An Affiliation of Ilona Kurnatowska is added: Affiliation 2: Department of Internal Medicine and Transplant Nephrology, Medical University of Lodz, Kopcinskiego 22, 90-549 Lodz, Poland.

**Author Contributions:** P.P.-G., contributed to the study design and writing the article; I.K., contributed to the study design; I.M.-B., contributed to the study design; M.P., contributed to the study design and writing the article, and conducted the statistical analysis and interpreted the data. All authors have read and agreed to the published version of the manuscript.
