# Supplementary material for: Twenty-Year Mortality Trends in Patients with Kidney Disease in Poland with the Use of the Years of Life Lost Measure, 2000–2019
Source: Int J Environ Res Public Health. 2022 Feb 24;19(5):2649. doi: 10.3390/ijerph19052649 (PMC8909903; doi:10.3390/ijerph19052649)
Supplement: Supplementary file 1 [file ijerph-19-02649-s001.zip › ijerph-1562024-supplementary.pdf]

Suppl. Number of deaths in patients with kidney disease in Poland in 2000-2019 according to cause (ICD-10 codes)

Causes of death	2000	2001	2002	2003	2004	2005	2006	2007	2008	2009	2010	2011	2012	2013	2014	2015	2016	2017	2018	2019	Total
N00 - Acute nephritic syndrome	6	11	9	4	7	8	6	5	6	5	11	2	4	14	15	44	106	119	138	258	778
N01 - Rapidly progressive nephritic syndrome	3	9	5	5	8	5	3	6	5	10	1	5	2	2	5	7	7	6	9	9	112
N02 - Recurrent and persistent haematuria	2	2	2	2		4		1	2					1	1	1	2		1	3	24
N03 - Chronic nephritic syndrome	87	95	72	64	47	37	31	36	17	19	23	26	70	57	54	182	295	380	520	633	2745
N04 - Nephrotic syndrome	22	17	14	15	29	12	23	20	10	19	13	16	22	36	23	16	28	31	49	55	470
N05 - Unspecified nephritic syndrome	32	38	28	22	30	35	32	21	19	20	17	10	15	11	3	10	7	18	60	6	434
N06 - Isolated proteinuria with specified morphological lesion			1	1	1		1	1							1		1				7
N07 - Hereditary nephropathy, not elsewhere classified					1																1
N10 - Acute tubulo-interstitial nephritis	14	14	19	17	17	14	30	49	51	63	86	81	114	121	110	105	85	62	122	137	1311
N11 - Chronic tubulo-interstitial nephritis	291	234	196	176	148	141	128	80	64	39	40	19	35	791	1230	1503	1373	1291	1371	1261	10411
N12 - Tubulo-interstitial nephritis, not specified as acute or chronic	10	4	10	7	8	17	18	8	5	14	14	12	21	9	12	20	11	11	31	25	267
N13 - Obstructive and reflux uropathy	33	43	44	48	37	29	46	36	35	38	41	47	51	59	65	50	60	88	89	81	1020
N14 - Drug- and heavy-metal-induced tubulo-interstitial and tubular conditions			1	3		1	1	1		1			1	3				3		4	19
N15 - Other renal tubulo-interstitial diseases	17	14	14	16	16	15	15	11	10	2	13	19	762	417	81	17	23	31	58	21	1572
N17 - Acute renal failure	256	262	271	285	369	412	553	582	772	1095	1180	1177	881	474	86	21	113	4	3	1	8797
N18 - Chronic kidney disease	2081	2180	2219	2305	2350	2621	2653	3003	3095	3004	3050	3270	2288	1580	309	103	203	17	21	57	36409
N19 - Unspecified kidney failure	996	890	875	848	881	911	885	596	508	541	477	299	252	130	31	5	11			5	9141
N20 - Calculus of kidney and ureter	46	49	37	32	23	34	25	21	11	15	9	15	12	18	25	22	21	31	54	34	534
N21 - Calculus of lower urinary tract	6	1	2	1	2	1	2	2			1	1	1	1	1	1	2	6	3	5	39
N23 - Unspecified renal colic					1	1															2
N25 - Disorders resulting from impaired renal tubular function	1	1		1		1	1			1	1		1	1	1	3	2	1	2	3	21
N26 - Unspecified contracted kidney	13	6	4	2	3	1	5	3	5	2	4	1					1				50
N27 - Small kidney of unknown cause			1			1		1			2							2			7
N28 - Other disorders of kidney and ureter, not elsewhere classified	30	22	14	22	21	8	15	30	14	7	6	30	45	9	11	6	198	446	380	194	1508
N39 - Other disorders of urinary system	48	53	61	70	105	163	155	53	70	103	114	151	349	501	699	841	910	1285	1627	1632	8990
Q61 - Cystic kidney disease	39	28	27	32	28	26	21	20	16	24	20	26	36	41	20	33	21	31	37	29	555
Total	4033	3973	3926	3978	4132	4498	4649	4586	4715	5022	5123	5207	4962	4276	2783	2990	3480	3863	4575	4453	85224
